# Cytomegalovirus Seroprevalence in Pregnant Women and Association with Adverse Pregnancy/Neonatal Outcomes in Jiangsu Province, China

**DOI:** 10.1371/journal.pone.0107645

**Published:** 2014-09-11

**Authors:** Shu Zhang, Lingqing Hu, Jie Chen, Biyun Xu, Yi-Hua Zhou, Yali Hu

**Affiliations:** 1 Department of Obstetrics and Gynecology, Nanjing Drum Tower Hospital, Nanjing University Medical School, Jiangsu, China; 2 Department of Obstetrics and Gynecology, Wuxi Maternal and Child Health Hospital, Jiangsu, China; 3 Department of Biostatistics, Nanjing Drum Tower Hospital, Nanjing University Medical School, Jiangsu, China; 4 Departments of Experimental Medicine and Infectious Diseases, Nanjing Drum Tower Hospital, Nanjing University Medical School, Jiangsu, China; 5 Jiangsu Key Laboratory for Molecular Medicine, Nanjing University Medical School, Jiangsu, China; University of British Columbia, Canada

## Abstract

**Background:**

In this study, we aimed to determine the provincial population-based seroprevalence in pregnant women and to further explore the association of maternal CMV infection status and adverse pregnancy/neonatal/growth outcomes in Jiangsu, China.

**Methods:**

In this case-control study, the sera from 527 pregnant women with adverse pregnancy/neonatal outcomes and 496 mothers of healthy infants in Jiangsu Province, collected at gestation age of 15–20 weeks, were tested for anti-CMV IgG, IgM and IgG avidity. Adverse pregnancy/neonatal outcomes were identified based on pregnancy/neonatal outcomes.

**Results:**

The overall seroprevalence of anti-CMV IgG was 98.7%, with 99.4% and 98.0% in the case and control groups, respectively (*P* = 0.039). The prevalence of anti-CMV IgG+/IgM+, was higher in the case group than that in the control group (3.8% vs. 1.6%, *P* = 0.033). Anti-CMV IgG avidity assay showed that none in the control group were primarily infected, but five (0.9%) in the case group underwent primary infection (*P* = 0.084); all five infants of these women presented severe adverse neonatal/growth outcomes. Exact logistic regression analysis showed that anti-CMV IgG+/IgM+ was associated with adverse pregnancy/neonatal/growth outcomes (aOR = 2.44, 95% CI 1.01–6.48, P = 0.047). Maternal low education level and prior abnormal pregnancies also were risk factors for adverse pregnancy/neonatal outcomes.

**Conclusions:**

In populations with very high prevalence of latent CMV infection, active maternal CMV infection during pregnancy might be a risk factor for adverse pregnancy/neonatal outcomes.

## Introduction

Human cytomegalovirus (CMV) infection is distributed throughout the world. It is well documented that the seroprevalence of CMV in pregnant women is 42.3–68.3% in developed countries [1], [2], but over 95% in developing countries such as China [3]–[5]. However, the data were mostly derived from hospital-based pregnant women in cities [6], [7], and it may not be representative of the general population from both urban and rural areas. Jiangsu Province is located in the east of China, and has the densest population with more than 76 million inhabitants. The seroprevalence in pregnant women based on a province-wide survey still needs to be defined.

Congenital CMV infection, occurring in approximately 1% of all live births [8], [9], can cause birth defects and childhood disabilities. Studies demonstrate that maternal primary CMV infection during pregnancy is the major cause of severe congenital infection in developed countries. Moreover, reactivation of latent infection or re-infection of different virus strain may also cause a high proportion of CMV congenital infection even in developed countries [10]. On the other hand, in developing countries with higher CMV seroprevalence, most congenitally infected neonates are born to women with recurrent infection during pregnancy, and the majority of infants are asymptomatic [11]. However, population-based study on the seroprevalence of CMV in pregnant women and the association with adverse pregnancy/neonatal outcomes is less reported.

In the current study based on a population of 19 904 pregnant women in Jiangsu, we determined the seroprevalence of anti-CMV IgG in pregnant women, and identified maternal CMV infection status in the case and control groups to evaluate the association of maternal CMV infection status and adverse pregnancy/neonatal outcomes.

## Methods

### Subjects and serum specimens

During August 2002 to July 2004, in a study on the provincial prevalence of birth defects in Jiangsu Province, China, 20 communities from 6 cities (urban) and 54 communities from 8 counties (rural areas) were selected as surveillance spots by stratified cluster sampling. All pregnant women at the first trimester from these areas were enrolled and serum samples from 19 904 pregnant women at 15–20 weeks of gestation were collected and kept at –30°C [12]. These women had been selected to represent the pregnant women population in Jiangsu, and they all delivered their infants in hospitals. General characteristics of each pregnant woman including maternal age, educational level and family income, gravidity and parity and history of abnormal pregnancies, were collected using a structured standard questionnaire. The obstetrical and perinatal information was recorded in the computer database by trained obstetricians [12]. Furthermore, each neonatal outcome was closely followed up: for a woman experienced fetus/infant loss, the cause was recorded; for a woman with live birth, the child’s health condition at the age of 4 years was also evaluated by qualified physicians [12]. Of the 19 904 pregnant women, 717 (3.6%) were not followed up, mainly because of refusal and address changes. Finally, 19 187 (96.4%) pregnant women with known pregnancy/neonatal outcomes were included in the current study.

In the current study, based on perinatal outcomes and child’s health conditions examined in the follow-up at four years old, adverse pregnancy/neonatal/growth outcomes were defined as the presence of one of any severe sequelae (Table 1). These included pregnancy loss, fetal, perinatal or infant death, congenital malformation, and intrauterine growth restriction or infant growth retardation, and others. However, induced abortion due to unwilling pregnancy and fetal/neonatal loss due to an accident were not included. A total of 527 singleton infants with adverse pregnancy/neonatal/growth outcomes were identified and designated as the case group (Table 1). Meanwhile, a same number of healthy infants in each area were selected as the control subjects using stratified random sampling. However, totally 34 serum samples from the randomly selected pregnant women with normal pregnancy outcomes were not available. Finally, the mothers of 496 singleton healthy infants were included in the control group.

**Table 1:** 527 singleton fetuses/infants with adverse pregnancy/neonatal/growth outcomes designated as the case group, out of 19 187 pregnant women.

Adverse pregnancy/neonatal/growth outcome	Number	Prevalence (%)
Spontaneous abortion	134	0.70
Pregnancy termination for fetal malformation	32	0.17
Fetal death*	32	0.17
Stillbirth†	7	0.04
Neonatal death	40	0.21
Infant death	29	0.15
Live birth with congenital anomaly§	68	0.35
Intrauterine growth restriction	129	0.67
Growth retardation¶	28	0.15
Mental retardation/dementia	26/2	0.15
Total	527	2.75

*Spontaneous intrauterine death of a fetus from gestation week 20 but before delivery.

† Fetal/infant death during delivery, which was not associated with improper management.

§ Including symptomatic abnormalities at birth and delayed or progressive abnormalities at follow-up, such as congenital heart diseases (12), congenital hydrocephalus and cerebral palsy (6), skull deformity (2), facial deformity (lip/cleft palate, etc. 15), strabismus (2) and amblyopia (3), dysaudia or hearing loss (4), congenital esophageal atresia (1) and megacolon (2), skeletal deformities (spine curvature, thoracic deformity, hand/foot deformity, etc. 17), and others (4).

¶ Identified based on child’s weight or height of less than two standard deviations below the mean for age, sex and district.

This study was performed according to the Declaration of Helsinki and approved by ethics committee of Jiangsu Family Planning Institute and Nanjing Drum Tower Hospital. Since the women consented to participate in the birth defect study conducted in 2002–2004 [12], their serum samples were used in the current study via an exemption approved by the institutional review boards of Jiangsu Family Planning Institute (EC2008501) and Nanjing Drum Tower Hospital (ECXK200709). In the follow-up study, all the women provided their written informed consent; mothers/fathers or guardians on the behalf of their children signed the written informed consent to participate in this study.

### Serological tests

All the serum samples were tested by a quantitative enzyme-linked immunosorbent assay (ELISA) kit (Dia.Pro Diagnostic Bioprobes, Milano, Italy) for anti-CMV IgG and a captured ELISA kit for anti-CMV IgM (Bell Biological Technology, Beijing, China) according to the manufacturer’s instructions. The maternal CMV infection status was defined based on the results in testing anti-CMV IgG and IgM. The women showing anti-CMV IgG positive but IgM negative were considered to be latent infection, and those showing positive for both anti-CMV IgG and IgM were defined to be active infection.

The women with positive anti-CMV IgG were further tested for anti-CMV IgG avidity index (AI) since anti-CMV IgG avidity has proven to be a powerful tool for distinguishing primary form non-primary infection [13]–[15]. Based on the other investigators’ results [16], [17] and our previous observations in subjects with documented seroconversion [18], [19], the test for anti-CMV IgG avidity shows the high sensitivity and specificity. Anti-CMV IgG avidity was determined by 8 M urea denaturation procedures using anti-CMV IgG ELISA kit (Dia.Pro Diagnostic Bioprobes) as previously described [18], [19]. For a given serum, an AI≤30% was considered as low, between 30% and 50% as intermediate, and greater than 50% as high avidity antibodies. Positive tests of both anti-CMV IgM and IgG with the low IgG avidity (AI≤30%) indicate a recent primary infection, while positive test of anti-CMV IgG alone with the intermediate to high IgG avidity indicates latent infection. In the test of anti-CMV IgM, IgG, and IgG avidity, we arranged the serum samples to be measured blindly, i.e., the investigators responsible for the testing were unaware of the serum identity; the sample identity was not known to them until the completion of all tests.

### Statistical analysis

Data analysis was performed using SPSS 13.0 statistical software. Continuous variables normally distributed were reported as mean ± standard deviation and compared by *t*-test between case and control groups; categorical variables were expressed as number and percentage and compared by *χ* ^2^ test or Fisher’s exact test where appropriate. Multivariate exact logistic regression analyses were further performed to determine the independent association of maternal CMV infection status (classified as active infection, latent infection, and without infection) and the adverse pregnancy/neonatal/growth outcomes, with pregnancy/neonatal/growth outcome as the binary dependent variable and maternal CMV infection status and other variables different between the case and control groups as the independent variables. The results were expressed by the adjusted odds ratios (aOR) with 95% confidence intervals (CI). A two-sided P value <0.05 is considered statistically significant.

## Results

### General characteristics

A total of 1023 mother-fetus/newborn pairs, 527 in case group and 496 in control group, were included in the current study. The pregnant women were 20–42 years old (mean, 25.1±3.3), and 9.2% (94/1023) of them were multiparas. Compared with the pregnant women in the case group, the women in the control group had similar ages, races, parity, levels of family income, and abnormal conditions during early pregnancy including vaginal bleeding, drugs intake, and toxic substance/X-ray exposure (Table 2). However, more women in the case group were illiterate or only received basic education (73.6% vs. 67.3%, *P* = 0.027), and more women in the case group were recorded with the history of adverse pregnancy/neonatal outcomes including intrauterine fetal death, stillbirth, congenital malformation, and spontaneous abortion twice or more (6.6% vs. 2.4%, *P* = 0.001).

**Table 2:** General characteristic of pregnant women with or without adverse pregnancy/neonatal/growth outcomes.

Characteristic	Case groupn = 527 (%)	Control groupn = 496 (%)	*P* value
Age (year)	26.0±3.9	25.3±3.3	0.379
Weight (kg)	56.2±8.1	56.4±7.5	0.717
Race			
Han	522 (99.1)	492 (99.2)	>0.99
Others	5 (0.9)	4 (0.8)	
Multipara	54 (10.2)	40 (8.1)	0.227
Education level*			
Low	388 (73.6)	334 (67.3)	0.027
High	139 (26.4)	162 (32.7)	
Income (¥/year)			
<18,000	159 (30.1)	146 (29.4)	0.313
18,000–36, 000	258 (49.0)	227 (45.8)	
>36, 000	110 (20.9)	123 (24.8)	
Maternal basic disease†	6 (1.1)	1 (0.2)	0.151
Previous adverse pregnancy outcomes§	35 (6.6)	12 (2.4)	0.001
Abnormalities in early pregnancy			
Contacting toxic substance/X-ray	16 (3.0)	17 (3.4)	0.723
Vaginal bleeding	58 (11.0)	51 (10.3)	0.708
Flu-like symptoms	36 (6.8)	26 (5.2)	0.287

*Low education level means illiteracy or basic education only, namely the Chinese nine-year compulsory education (elementary school education for 6 years and junior high school education for 3 years). Senior high school education and above was considered as “high level”.

† Maternal basic disease: maternal disease existing before conception, including hypertension, cardiac disease, systematic lupus erythematosus and hyperthyreosis in this study.

§ Prior adverse pregnancy outcomes: including spontaneous abortion for twice or more, previous fetal, neonatal or infant death, congenital malformation, and growth retardation.

### Seroprevalence of CMV in pregnant women

Of the 1023 pregnant women, 13 (1.3%) were tested negative for both anti-CMV IgG and IgM, and none showed anti-CMV IgG negative but IgM positive. The overall prevalence of anti-CMV IgG was 98.7% (1010/1023). The seroprevalence (524/527, 99.4%; 95% CI 98.79–100%) in the case group was comparable with that (98.0%, 486/496; 95% CI 96.75–99.22%) in the control group, although the difference was statistically significant (P = 0.039). Based on the criteria for interpreting maternal CMV infection status [15], the prevalence of solely anti-CMV IgG+ was not statistically different between the two groups (504/527, 95.6% vs. 478/496, 96.4%, *P* = 0.549). However, the prevalence of anti-CMV IgG+/IgM+, which indicates active infection, was higher in the case group (20/527, 3.8%) than that in the control group (8/496, 1.6%, *P* = 0.033). Furthermore, we determined the IgG avidity in all 28 anti-CMV IgG+/IgM+ women to define the maternal CMV infection status. The results displayed that 15 (2.8%) women in the case group and 8 (1.6%) women in the control group were non-primarily infected respectively (*P* = 0.184); the 5 (0.9%) other women in the case group underwent primary infection during pregnancy, but none of the women in the control group were primarily infected during pregnancy (*P* = 0.084). Additionally, the CMV seroprevalence showed no significant difference between urban and rural communities in the case (urban 184/185, 99.5% vs. rural 340/342, 99.4%) or control (135/138, 97.8% vs. 351/358, 98.0%) group.

### Risk factors for adverse pregnancy/neonatal/growth outcomes

The current study showed that the prevalence of anti-CMV IgG+/IgM+ in the case group was higher than that in the control group (3.8% vs 1.6%, *P* = 0.033). Importantly, all five infants born to the mothers with primary infection during pregnancy had adverse pregnancy/neonatal/growth outcomes, including one case with neonatal death, two with growth retardation, one with skull deformity, and one with purulent meningitis (Table 3). Table 3 also shows the pregnancy/neonatal/growth outcomes or the clinical information on the infants of 15 mothers with anti-CMV IgG AI >30% in the case group and on the infants of 8 mothers in the control group.

**Table 3:** Pregnancy/neonatal outcomes and child growth in mothers with active CMV infection during pregnancy.

	CMV IgM	CMV IgG AI (%)	Neonatal parameter				Pregnancy/neonatal/growth outcome
			Sex	Birth weight	Birth height	Apgar score	
CMV IgG AI≤30%							
1	+	28.6	F	2600	47	0	Neonatal death
2	+	27.6	M	3400	50	10	Growth retardation
3	+	23.8	M	2900	50	10	Growth retardation
4	+	23.0	F	3000	48	10	Skull deformity
5	+	22.4	M	3000	50	9	Purulent meningitis
CMV IgG AI>30%							
6	+	31.9	–	–	–	–	Spontaneous abortion
7	+	32.3	–	–	–	–	Spontaneous abortion
8	+	32.9	M	3520	50	9	Dementia, deafness, and mute
9	+	34.7	M	4400	58	10	Flaccid paralysis
10	+	36.8	–	–	–	–	Spontaneous abortion
11	+	39.4	–	–	–	0	Fetal death
12	+	40.1	M	3000	50	10	Neonatal death (cleft lip and palate)
13	+	42.2	F	3500	50	10	Growth retardation
14	+	44.8	M	3150	50	10	Mild hearing loss (left ear)
15	+	48.1	F	3250	50	10	Growth retardation
16	+	50.7	–	–	–	0	Stillbirth (gastroschisis)
17	+	51.8	M	3700	50	10	Strabismus (left eye) and amblyopia
18	+	53.1	–	–	–	–	Spontaneous abortion
19	+	56.5	–	–	–	–	Induced labor (fetal growth restriction)
20	+	88.0	–	–	–	–	Induced abortion (embryo withering)
21	+	31.0	M	3600	50	10	Normal
22	+	33.0	F	3800	49	10	Normal
23	+	33.9	M	3500	53	10	Normal
24	+	40.5	F	3500	50	10	Normal
25	+	41.9	M	3500	43	9	Normal
26	+	43.8	F	3500	51	10	Normal
27	+	44.9	M	2750	48	10	Normal
28	+	48.6	M	3300	50	9	Normal

We further performed the exact logistic regression analysis after adjusting for variables between the two groups (Table 2). The results indicated that, after controlling for potential confounding factors including educational level and history of abnormal pregnancies, maternal active infection during pregnancy was associated with an increased risk for adverse pregnancy/neonatal/growth outcomes (aOR = 2.44, 95% CI 1.01–6.48, P = 0.047). However, when the adverse outcomes in the presumed recurrent infection and primary infection were separately compared to those in the latent infection, the exact logistic regression analysis showed that, although the aORs were 1.825 (95% CI 0.714–5.045) and 6.505 (95% CI 0.882–∞) respectively, neither of them had statistical significance (*P* = 0.246 and 0.069 respectively). On the other hand, low education level (aOR = 1.35, 95% CI 1.01–1.78, P = 0.0419) and prior abnormal pregnancies (aOR = 2.92, 95% CI 1.49–5.74, P = 0.0018) were also independently associated with an increased risk for adverse pregnancy/neonatal/growth outcomes.

## Discussion

In the current study, the seroprevalence of anti-CMV IgG in the pregnant women with adverse pregnancy/neonatal/growth outcomes was slightly higher than that in the mothers of healthy infants, however, the women with adverse pregnancy/neonatal outcomes accounted for a low percentage (527/19187, 2.75%) of the whole pregnant woman population. Additionally, the 496 women in the control group were stratified randomly selected from the pregnant women enrolled, who had been selected as a good representative of the whole pregnant women population in Jiangsu. Therefore, based on the seroprevalence of pregnant women in the control group, the provincial seroprevalence of CMV in pregnant women was as high as 98.0%, with 96.4% of latent infection and 1.6% of active infection. Our findings from the pregnant women in Jiangsu province, having comparable economical status and residential density, were in accordance with the previous study conducted in Beijing, China, where the prevalence of anti-CMV IgG and IgM in 4692 childbearing-age or pregnant women was 92.5% and 1.8% respectively [6]. The seroprevalence in the current study is also comparable to that (96.4%) in pregnant women from Nanjing, the capital of Jiangsu province [19]. Thus, the high seroprevalence indicates that CMV infection is now universal in Chinese pregnant women. The lifestyle involving crowded living status, poor childbearing practice, and weak awareness of taking hygienic measures may contribute to this situation [20].

Numazaki et al [21] reported that, in the last two decades, the seroprevalence of CMV in pregnant women from Japan has substantially decreased from 85% to 68.1%. But in our country, compared with the previous prevalence of 94.0–95.6% [22]–[24], recent studies showed comparable seroprevalence in women at childbearing age despite notable advancement in public health care system [6], [7], [20]. It may be related to the fact that primary CMV infection mostly occurs early in childhood [19], and poor economy and public health during their infancy is likely to attribute to the high seroprevalence in women of childbearing age. On the other hand, the current seroprevalence in young children in Nanjing was approximately 80% [19], much lower than the current 95–98% of the childbearing age women, indicating the effectiveness of public health improvement in reducing the incidence of infectious diseases in China.

In the current study, only a small proportion (3.8%, 20/527) of the women with the adverse pregnancy/neonatal/growth outcomes experienced active CMV infection during pregnancy. This may be associated with very high proportion of pregnant women with preconceptional immunity to CMV since they were positive for anti-CMV IgG with the high IgG avidity. This is in agreement with the previous studies [15], [25], [26]. The preconceptional maternal immunity may provide substantial protection against the vertical transmission to the fetus and decreases the risk of symptomatic congenital infection [27].

Maternal primary CMV infection during pregnancy is more likely to result in congenital CMV infection with severe neonatal morbidity and mortality [28]. In the current study, based on the anti-CMV positivity and low anti-CMV IgG avidity, we defined the primary CMV infection in five pregnant women, who all happened to have adverse pregnancy/neonatal/growth outcomes (Table 3). This appears to be somewhat inconsistent with the usual 40% transmission and 10% disease rate in infants of mothers with primary CMV infection in pregnancy. Since all laboratory tests were carried out in a blind manner, the results should reflect the facts. However, because the current investigation was retrospective and confirmatory tests for CMV in the infants/children were infeasible to perform, whether all of these five infants/children were congenitally infected with CMV is uncertain. Similarly, it was hard to clarify whether the infants/children of 15 mothers with presumed recurrent infection in the case group and of 8 mothers in the control group were congenitally infected with CMV. It should be emphasized that no statistical significant association between primary or presumed recurrent infection and the adverse pregnancy/neonatal outcomes in the exact logistic regression analysis is very likely caused by the limited number of subjects with active CMV infection.

Noticeably, the anti-CMV IgM seroprevalence of current study (3.8% in the case group and 1.6% in the control group) is similar to that in Beijing [6], but is lower than the prevalence of 5.6–8.8% previously reported in China [22]–[24]. The difference might be attributed to two reasons. One may be the different study subjects; all the three reports included the pregnant women from the hospitals [22]–[24], but the current study included the women based on the population. The other may be related to different assays for anti-CMV IgM; the assays used in the three reports were indirect ELISA, whereas that used in the current study was captured ELISA. It is generally considered that, for detecting IgM, captured ELISA has higher specificity than indirect ELISA.

There are some limitations in the current study. First, we could not make a definite diagnosis of congenital infection due to the lack of fetal/infants’ specimens, leading to the failure to establish the causal relationship between CMV infections during pregnancy and the adverse outcomes. Second, the diagnosis of CMV infection status in the pregnant women is not all definitely confirmed. A recurrent CMV infection should be based on an increase (4-fold or more) of IgG titers in follow-up serum sample(s), and a latent infection should be defined in pregnant women with stable titers of IgG with high avidity in the follow-up. But we could not follow up the patients since our study was retrospective. Third, there may be other maternal characteristics or even unknown risk factors associated with adverse pregnancy/neonatal outcomes.

In view of these results, we conclude that, in mainland China, CMV infection is ubiquitous in pregnant women and the vast majority of women of childbearing age have acquired natural immunity to CMV before pregnancy. Thus, it is likely that, in highly immune populations, most cases of congenital infection are a result of maternal presumed recurrent infection. Before deciding whether to routinely screen active CMV infection in the pregnant women in countries with high anti-CMV IgG seroprevalence, further investigations are required to determine the proportion of congenital CMV infections in the overall fetal malformations and adverse neonatal outcomes.

## References

[1] De Paschale M, Agrappi C, Manco MT, Paganini A, Clerici P (2009) Incidence and risk of cytomegalovirus infection during pregnancy in an urban area of Northern Italy. Infect Dis Obstet Gynecol 2009: 206–505. 10.1155/2009/206505 PMC2715896 19639052

[2] Enders G, Daiminger A, Lindemann L, Knotek F, Bäder U, et al (2012) Cytomegalovirus (CMV) seroprevalence in pregnant women, bone marrow donors and adolescents in Germany, 1996–2010. Med Microbiol Immunol 201: 303–309. 22398714 10.1007/s00430-012-0232-7

[3] Uysal A, Taner CE, Cüce M, Atalay S, Göl B, et al (2012) Cytomegalovirus and rubella seroprevalence in pregnant women in Izmir/Turkey: follow-up and results of pregnancy outcome. Arch Gynecol Obstet 286: 605–608. 22546952 10.1007/s00404-012-2353-z

[4] Yamamoto AY, Castellucci RA, Aragon DC, Mussi-Pinhata MM (2013) Early high CMV seroprevalence in pregnant women from a population with a high rate of congenital infection. Epidemiol Infect 141: 2187–2191. 23200458 10.1017/S0950268812002695 PMC9151393

[5] Neirukh T, Qaisi A, Saleh N, Abu Rmaileh A, Abu Zahriyeh E, et al (2013) Seroprevalence of Cytomegalovirus among pregnant women and hospitalized children in Palestine. BMC Infect Dis 13: 528. 24206533 10.1186/1471-2334-13-528 PMC3830538

[6] Li Z, Yan C, Liu P, Yan R, Feng Z (2009) Prevalence of serum antibodies to TORCH among women before pregnancy or in the early period of pregnancy in Beijing. Clin Chim Acta 403: 212–215. 19302994 10.1016/j.cca.2009.03.027

[7] Fang FQ, Fan QS, Yang ZJ, Peng YB, Zhang L, et al (2009) Incidence of cytomegalovirus infection in Shanghai, China. Clin Vaccine Immunol 16: 1700–1703. 19776197 10.1128/CVI.00385-08 PMC2772370

[8] Kourí V, Correa CB, Verdasquera D, Martínez PA, Alvarez A, et al (2010) Diagnosis and screening for cytomegalovirus infection in pregnant women in Cuba as prognostic markers of congenital infection in newborns: 2007–2008. Pediatr Infect Dis J 29: 1105–1110. 20622711 10.1097/INF.0b013e3181eb7388

[9] Yamamoto AY, Mussi-Pinhata MM, Isaac Mde L, Amaral FR, Carvalheiro CG, et al (2011) Congenital cytomegalovirus infection as a cause of sensorineural hearing loss in a highly immune population. Pediatr Infect Dis J 30: 1043–1046. 21814153 10.1097/INF.0b013e31822d9640 PMC3222783

[10] Cannon MJ (2009) Congenital cytomegalovirus (CMV) epidemiology and awareness. J Clin Virol 46: S6–10. 19800841 10.1016/j.jcv.2009.09.002

[11] Ornoy A, Diav-Citrin O (2006) Fetal effects of primary and secondary cytomegalovirus infection in pregnancy. Reprod Toxicol 21: 399–409. 16580941 10.1016/j.reprotox.2005.02.002

[12] Zhou J, Hu Y, Liu Q, Chen Q, Xu B, et al (2007) Analysis of birth defects based on population survey in Jiangsu Province. Jiangsu Yi Yao 33: 1218–1220 (in Chinese).

[13] Munro SC, Hall B, Whybin LR, Leader L, Robertson P, et al (2005) Diagnosis of and screening for cytomegalovirus infection in pregnant women. J Clin Microbiol 43: 4713–4718. 16145132 10.1128/JCM.43.9.4713-4718.2005 PMC1234061

[14] Lazzarotto T, Guerra B, Lanari M, Gabrielli L, Landini MP (2008) New advances in the diagnosis of congenital cytomegalovirus infection. J Clin Virol 41: 192–197. 18054840 10.1016/j.jcv.2007.10.015

[15] Tabatabaee M, Tayyebi D (2009) Seroepidemiologic study of human cytomegalovirus in pregnant women in Valiasr Hospital of Kazeroon, Fars, Iran. J Matern Fetal Neonatal Med 22: 517–521. 19350446 10.1080/14767050902801678

[16] Revello MG, Gerna G (2002) Diagnosis and management of human cytomegalovirus infection in the mother, fetus, and newborn infant. Clin Microbiol Rev 15: 680–715. 12364375 10.1128/CMR.15.4.680-715.2002 PMC126858

[17] Enders G, Daiminger A, Bäder U, Exler S, Schimpf Y, et al (2013) The value of CMV IgG avidity and immunoblot for timing the onset of primary CMV infection in pregnancy. J Clin Virol 56: 102–107. 23153820 10.1016/j.jcv.2012.09.015

[18] Wu S, Hu Y, Zhang S, Zhou YH (2009) Evaluation of cytomegalovirus IgG avidity index in differentiation of primary infection from reactivation. Lin Chuang Jian Yan Za Zhi 27: 217–219 (in Chinese).

[19] Chen J, Hu L, Wu M, Zhong T, Zhou YH, et al (2012) Kinetics of IgG antibody to cytomegalovirus (CMV) after birth and seroprevalence of anti-CMV IgG in Chinese children. Virol J 9: 304. 23228149 10.1186/1743-422X-9-304 PMC3544651

[20] Chen MH, Chen PC, Jeng SF, Hsieh CJ, Su FC, et al (2008) High perinatal seroprevalence of cytomegalovirus in northern Taiwan. J Paediatr Child Health 44: 166–169. 17854407 10.1111/j.1440-1754.2007.01215.x

[21] Numazaki K, Fujkawa T (2002) Prevalence of serum antibodies to cytomegalovirus in pregnant women in Sapporo, Japan. Int J Infect Dis 6: 147–148. 12146500 10.1016/s1201-9712(02)90078-0

[22] Guo T (1992) Study of primary CMV infection in pregnant women. Zhonghua Liu Xing Bing Xue Za Zhi 13: 76–78 (in Chinese). 1327533

[23] Zhong XY, Ma TY (1993) A clinical study of cytomegalovirus infections during pregnancy. J Tongji Med Univ 13: 60–64. 8392114 10.1007/BF02886597

[24] Wu GJ, Chen SZ, Chen LY (2001) Study on molecular epidemiology of HCMV infection in mothers and their newborns in Changsha. Hunan Yi Ke Da Xue Xue Bao 26: 23–25 (in Chinese). 12536607

[25] Mussi-Pinhata MM, Yamamoto AY, Moura Brito RM, de Lima Isaac M, de Carvalho e Oliveira PF, et al (2009) Birth prevalence and natural history of congenital cytomegalovirus infection in a highly seroimmune population. Clin Infect Dis 49: 522–528. 19583520 10.1086/600882 PMC2778219

[26] Bagheri L, Mokhtarian H, Sarshar N, Ghahramani M (2012) Seroepidemiology of cytomegalovirus infection during pregnancy in Gonabad, east of Iran: a cross-sectional study. J Res Health Sci 12: 38–44. 22888713

[27] Lazzarotto T, Guerra B, Gabrielli L, Lanari M, Landini MP (2011) Update on the prevention, diagnosis and management of cytomegalovirus infection during pregnancy. Clin Microbiol Infect 17: 1285–1293. 21631642 10.1111/j.1469-0691.2011.03564.x

[28] Johnson JM, Anderson BL (2013) Cytomegalovirus: should we screen pregnant women for primary infection? Am J Perinatol 30: 121–124. 23292913 10.1055/s-0032-1333133

